# Rifaximin for Irritable Bowel Syndrome: A Meta-Analysis of Randomized Placebo-Controlled Trials

**DOI:** 10.1097/MD.0000000000002534

**Published:** 2016-01-29

**Authors:** Jun Li, Wenhua Zhu, Wenhui Liu, Yingqiao Wu, Benyan Wu

**Affiliations:** From the Department of Gastroenterology, Chinese PLA General Hospital, Fuxing Road (JL, WL, YW, BW); and Department of Oncology, Chinese 309th Hospital of PLA, Hei Shan Hu Road, Beijing, China (WZ).

## Abstract

The current treatments for irritable bowel syndrome (IBS) are suboptimal. The findings of previous studies of rifaximin treatment for IBS may have differed due to variations in study design.

Our study aimed to determine the therapeutic and adverse effects of rifaximin treatment for IBS based on a meta-analysis of published randomized controlled trials (RCTs).

We searched the MEDLINE, EMBASE, EBSCO, Springer, Ovid, and Cochrane Library databases for RCTs investigating the effects of rifaximin on IBS. Data from each selected RCT was evaluated individually based on an intention-to-treat analysis, and a meta-analysis was performed in which the odds ratios (ORs) and 95% confidence intervals (CIs) of clinical outcomes and adverse events were calculated using fixed-effects models.

Four eligible studies were identified. Overall relief of IBS symptoms in the rifaximin groups was greater than that in the placebo groups at the ends of both the treatment and follow-up periods (OR = 1.19; 95% CI: 1.08–1.32 and OR = 1.36; 95% CI: 1.18–1.58, respectively, *P* < 0.05 for both). Significant relief of abdominal distention was observed at the follow-up endpoint (OR = 1.69; 95% Cl: 1.27–2.23; *P* < 0.05), but not at the treatment endpoint (OR = 1.19; 95% CI: 0.96–1.49; *P* > 0.05). Abdominal pain (OR = 1.01; 95% CI: 0.98–1.03; *P* > 0.05), nausea (OR = 1.00; 95% CI: 0.98–1.02; *P* > 0.05), vomiting (OR: 0.99; 95% CI: 0.98–1.01; *P* > 0.05), and headache (OR = 1.01; 95% CI: 0.98–1.03; *P* > 0.05) did not differ significantly between the rifaximin and placebo groups.

In the RCTs selected, our meta-analysis showed that the efficacy of rifaximin for the resolution of overall IBS symptoms was greater than that of the placebos, and that rifaximin was well-tolerated. The course of relief from abdominal distention in IBS patients treated with rifaximin may be delayed in some patients, compared with that of overall IBS symptom relief.

## INTRODUCTION

Irritable bowel syndrome (IBS) is characterized by chronic intermittent abdominal discomfort with accompanying diarrhea and/or constipation in patients for whom imaging, biochemical, and morphological abnormalities of the digestive system are absent.^1^ Patients with IBS suffer frequent recurrence, which impairs their quality of life. In recent years, the incidence of IBS has gradually increased to ∼20% in European and American countries and 10% in China.^2^ It is more common in women and people ≤ 50 years of age.^3,4^ Studies have linked IBS to altered intestinal flora, visceral hypersensitivity, dysfunctional gastrointestinal motility, stress-induced inflammation, defects in the brain-gut neuronal axis, and psychological factors.^5–10^ However, the physiological mechanism underlying the pathogenesis of IBS remains unclear.

No reliable therapies are currently available for IBS. Drugs used to treat IBS include antispasmodics, antidiarrheals, cathartic drugs, and antidepressants. A recent nonsystematic review suggested that a specific subtype of IBS, referred to as small intestinal bacterial overgrowth (SIBO), occurs secondary to an intestinal bacterial infection,^11^ and previous studies have shown that changes in the diversity of small intestinal bacteria^12^ were linked to the pathogenesis of IBS.^10,13–15^ Multiple mechanisms have been proposed to explain how an imbalance in the intestinal flora causes IBS-related symptoms, including fermentation,^16,17^ disruption of the mucosal barrier,^18^ altered gastrointestinal motility,^19^ and the inflammatory immune response.^20^ Traditional antibiotics, such as neomycin, have been effective for treating IBS caused by dysbacteriosis.^21^ However, antibiotics have not been used extensively to treat IBS because of the risks of side effects and the development of drug resistance.

Rifaximin is a rifamycin derivative that inhibits bacterial gene expression. The bioavailability of rifaximin is low because it is poorly absorbed in the digestive tract, which reduces the risk of serious side effects, such as systemic immune hypersensitivity. The poor absorption of rifaximin also aids in maintaining an effective concentration of the drug in the intestinal lumen,^22^ making it useful for treating intestinal bacterial infections, and rifaximin has been approved by the United States Food and Drug Administration for the treatment of traveler's diarrhea in certain patients.^23^ Multiple recent clinical trials have demonstrated the beneficial therapeutic effects of rifaximin for the treatment of IBS.^24–27^ We performed a meta-analysis of the results of these studies to comprehensively evaluate the efficacy and adverse effects of rifaximin treatment for IBS in an effort to provide more reliable evidence of its clinical benefits.

## METHODS

### Data Sources and Search Strategy

Our meta-analysis was performed according to the recommendations of the Cochrane Handbook,^28^ and this report was prepared according to the Preferred Reporting Items for Systematic Reviews and Meta-Analyses (PRISMA) guidelines.^29^ The Institutional Review Board of Chinese PLA General Hospital deemed our study to exempt from review because only public available data were included in our analysis. We searched for the MEDLINE, EMBASE, EBSCO, Springer, Ovid, and Cochrane Library databases for published reports of randomized controlled trials (RCTs) published in English that evaluated the effects of rifaximin on IBS clinical outcomes. The following keywords were used for our search: “rifaximin” [MeSH], “irritable bowel syndrome” [MeSH], “IBS” [MeSH], “diarrhea” [MeSH], and “randomized controlled trial” [MeSH]. We used a date range for our search ending in 2015. The References section of retrieved articles was also searched manually to identify other relevant RCTs.

### Study Selection

Two reviewers (JL and WHZ) independently reviewed the full text versions of all the articles retrieved in the literature search to identify eligible studies. Studies that met the following criteria were included in our analysis: (1) clinical trial; (2) included patients ≥18 years of age only; (3) described randomization in detail; (4) placebo-controlled study; (5) implemented allocation concealment; (6) blinding of patients and research staff; (7) appropriately recorded withdrawals; and (8) evaluated the effects of rifaximin on IBS at the ends of both the treatment and follow-up periods. Studies that met the following criteria or did not meet the inclusion criteria were excluded from our analysis: (1) joint therapy trials using both rifaximin and another medication; (2) included subjects suffering from severe enteric disease; or (3) included subjects who developed a malignancy, heart failure, or renal failure during the study period. No limitations were made based on the gender. Conflicts in study selection were resolved by a third reviewer (YQW and BYW).

### Study Appraisal and Data Extraction

Two reviewers (JL and WHL) used a standardized data extraction form to independently extract the data from the studies included in our meta-analysis. The following items comprised the extracted data: (1) general information, including publication year, name of first author, location of the study centers, trial duration, number of participants, and number of withdrawals; (2) details of the study design, including descriptions of the blinding, allocation concealment, randomization methods, and bias prevention; (3) description of the experimental and placebo treatment protocols; (4) clinical outcomes at the end of the treatment and follow-up periods; and (5) adverse events occurring during rifaximin treatment.

### Statistical Analysis

The results of the selected studies were evaluated individually subjected to an intention-to-treat analysis using the Origin, version 8.3, software (Origin Lab, Northampton, MA). The meta-analysis was performed using the Review Manager, version 5.2, software obtained via The Cochrane Collaboration website (http://tech.cochrane.org/revman). Random- or fixed-effects models were used in the meta-analysis to calculate the overall odds ratio (OR) and 95% confidence interval (CI) for each clinical outcome and adverse event reported. Heterogeneity in the clinical outcomes was evaluated based on the *I*^2^ statistic using a chi-squared analysis. A fixed-effects model was used to estimate risk when significant heterogeneity in the data was not detected (*I*^2^ ≤ 50%), whereas a random-effects model was used when significant heterogeneity in the data was detected (*I*^2^ > 50%). Forest plots were constructed based on the results of the risk analysis. The overall effect size was evaluated using a *Z* test, with a *P* value for *Z* <0.05 indicating a statistically significant difference.

## RESULTS

### Study Characteristics

Our search identified 108 records. After screening the titles and abstracts of these articles, 98 were considered ineligible based on the inclusion criteria. After reviewing the full-length journal articles, an additional 6 articles were excluded based on the exclusion criteria. The remaining 4 RCTs were selected for our meta-analysis (Table 1).^24–27^ These 4 RCTs had publication dates ranging from 2006 to 2011, and all of them were performed in North America. The selected RCTs included a total of 1803 participants who ranged in age from 18 to 45 years, the majority of whom were of white ethnicity (non-Hispanic Caucasian). A small proportion of African Americans and people of other ethnicities were also included in these studies. All of the selected RCTs reported the resolution of overall IBS symptoms at the end of the treatment and follow-up periods, and one of these studies also reported the relief of abdominal distention at the end of the treatment and follow-up periods.^25^ Methods of double blinding and randomization were adequately described in all of the selected RCTs, and allocation concealment was described adequately in 2 of these studies.^24,27^ Three of the selected RCTs reported adverse events, including abdominal pain, nausea, vomiting, and headache.^24,26,27^

**TABLE 1:**
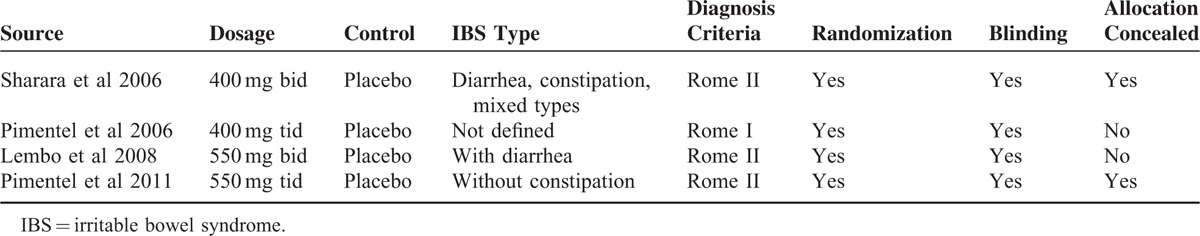
Summary of Study Characteristics

### Therapeutic Effects of Rifaximin

#### Overall Symptom Relief

**FIGURE 1:**
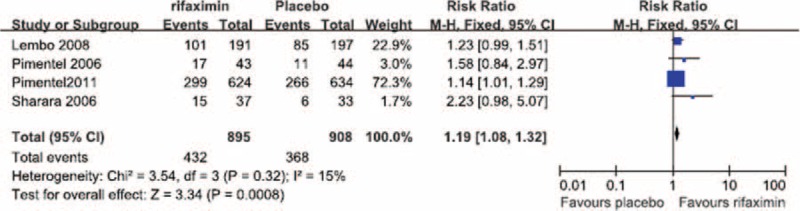
Comparison of overall symptom relief at the end of the treatment period.

**FIGURE 2:**
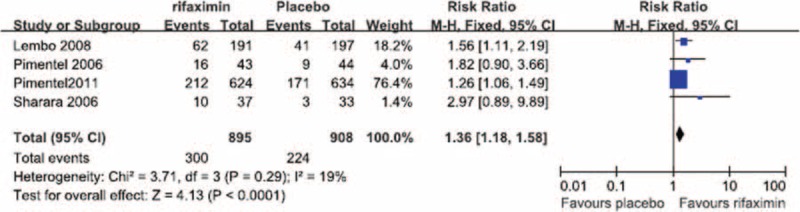
Comparison of overall symptom relief at the end of the follow-up period.

No significant heterogeneity was observed in the relief of overall IBS symptoms at the end of the treatment period (*I*^2^ = 15%). The fixed-effects model showed that, at the end of the treatment period, the remission of overall IBS symptoms was significantly greater in patients treated with rifaximin (OR = 1.19; 95% CI: 1.8–1.32), compared with that in patients treated with a placebo (*Z* = 3.34; *P* = 0.0008; Figure 1). No significant heterogeneity was observed in the relief of overall IBS symptoms at the end of the follow-up period (*I*^2^ = 19%). The fixed-effects model showed that, at the end of the follow-up period, the remission of overall IBS symptoms in the rifaximin groups was significantly greater (OR = 1.36; 95% CI: 1.18–1.58) than that in the placebo groups (*Z* = 4.13; *P* < 0.0001; Figure 2).

#### Resolution of Abdominal Distention

In the single study in which abdominal distention was assessed, no significant difference in abdominal distention was observed at the end of treatment period between the patients treated with rifaximin and those who received a placebo (OR = 1.19; 95% CI: 0.96–1.49; *Z* = 1.60; *P* = 0.11; Table 2). However, at the end of follow-up period, the fixed-effects model showed that the reduction in abdominal distention among patients treated with rifaximin was significantly greater (OR = 1.69; 95% CI: 1.27–2.23) than that in patients treated with a placebo (*Z* = 3.63, *P* = 0.0003; Table 2).

**TABLE 2:**
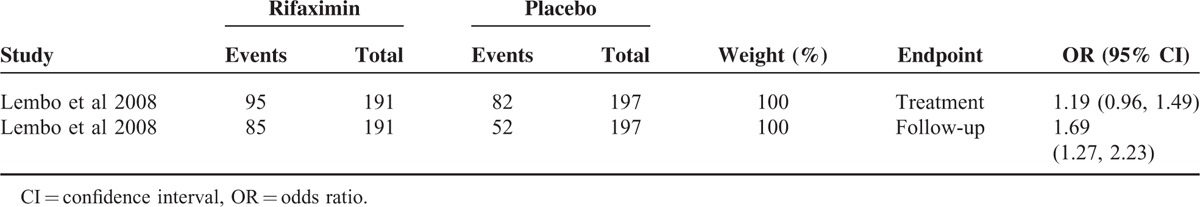
Comparison of Relief of Abdominal Distention at Different Time Points

### Adverse Effects of Rifaximin

#### Abdominal Pain

**FIGURE 3:**
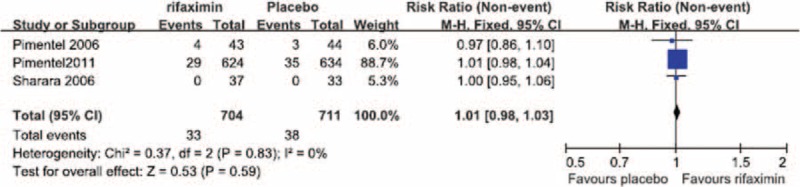
Comparison of abdominal pain.

No significant heterogeneity in the occurrence of abdominal pain during the treatment period was observed between the 3 RCTs in which abdominal pain was reported (*I*^2^ = 0%). The fixed-effect model showed that the risk of abdominal pain did not differ significantly between the patients treated with rifaximin (OR = 1.01; 95% CI: 0.98–1.03) and those who received a placebo (*Z* = 0.53, *P* = 0.59; Figure 3).

#### Nausea

**FIGURE 4:**
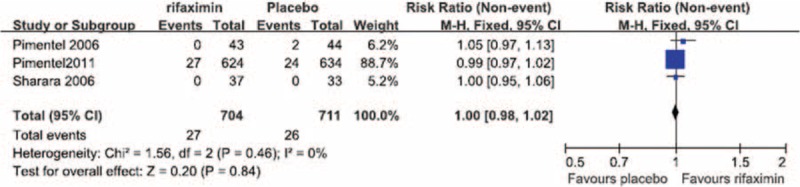
Comparison of nausea.

No significant heterogeneity in the occurrence of nausea during the treatment period was observed between the 3 RCTs in which nausea was reported (*I*^2^ = 0%). The fixed-effect model showed that the risk of nausea did not differ significantly between the patients treated with rifaximin (OR = 1.00; 95% CI: 0.98–1.02) and those who received a placebo (*Z* = 0.20, *P* = 0.84; Figure 4).

#### Vomiting

**FIGURE 5:**
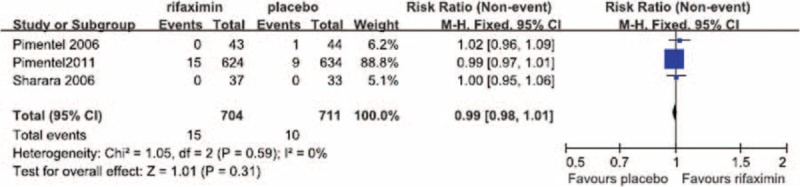
Comparison of vomiting.

No significant heterogeneity in the occurrence of vomiting during the treatment period was observed between the 3 RCTs in which vomiting was reported (*I*^2^ = 0%). The fixed-effect model showed that the risk of vomiting did not differ significantly between the patients treated with rifaximin (OR = 0.99; 95% CI: 0.98–1.01) and those who received a placebo (*Z* = 1.01, *P* = 0.31; Figure 5).

#### Headache

**FIGURE 6:**
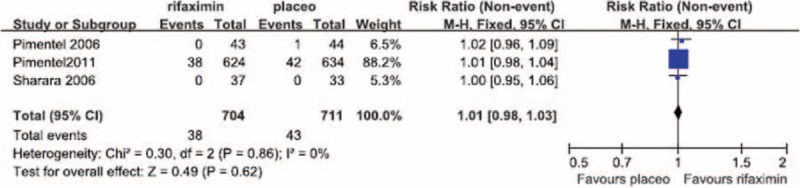
Comparison of headache.

No significant heterogeneity in the occurrence of headache during the treatment period was observed between the 3 RCTs in which headache was reported (*I*^2^ = 0%). The fixed-effect model showed that the risk of headache did not differ significantly between the patients treated with rifaximin (OR = 1.01; 95% CI: 0.98–1.03) and those who received a placebo (*Z* = 0.49, *P* = 0.62; Figure 6).

## DISCUSSION

Rifaximin is a broad-spectrum antibiotic that inhibits the beta subunit of bacterial DNA-dependent RNA polymerase and thus suppresses bacterial gene expression.^30^ Rifaximin is generally administered for the treatment of traveler's diarrhea and hepatic encephalopathy.^31,32^ Previous studies have reported significant improvement in IBS symptoms among patients with a positive SIBO-related lactulose hydrogen breath test before rifaximin treatment,^33,34^ which is consistent with direct evidence of the pharmacological mechanism of rifaximin in the treatment of IBS based on quantitative analysis of bacteria in the duodenal aspirate of IBS patients.^10^ Although the efficacy of rifaximin treatment for IBS has been demonstrated in multiple previous studies,^35^ inconsistencies among these studies have been noted regarding patient selection, clinical end points, and statistical analyses,^36^ and significant SIBO recurrence has been reported among IBS patients following rifaximin treatment.^37^

We performed a meta-analysis of RCTs that examined IBS clinical outcomes at the ends of both the treatment and follow-up periods to clarify the short-term and long-term therapeutic benefits of rifaximin treatment, respectively. Our meta-analysis of the selected studies showed the that, at the ends of both the treatment and follow-up periods, the likelihood of overall symptom relief was significantly greater in the rifaximin groups than in the placebo groups (Figures 1 and 2, respectively). In addition, although no significant difference in the relief of abdominal distention, a major symptom of IBS, was observed between the rifaximin and placebo groups at the treatment endpoint, the remission of abdominal distention in the rifaximin group was significantly greater than that of the placebo group at the follow-up endpoint (Table 2). We also investigated whether rifaximin treatment was associated with a greater risk of adverse effects. We found no significant difference in the risk of abdominal pain, nausea, vomiting, and headache between the rifaxmin and placebo groups at the treatment endpoint (Figures 3–6), demonstrating that rifaximin was well-tolerated.

Our findings our, however, subject to certain limitations. Our analyses of overall symptom relief and adverse effects included 4 and 3 studies, respectively, which limited the statistical power our results and rendered the use of funnel plots to evaluate publication bias impractical. In addition, our analysis of abdominal distention was based on the results of 1 study only, which may have confounded our results. In the studies included in our meta-analysis, the main parameters of efficacy were based on self-reported symptom relief and were therefore subjective in nature. Previous studies have shown that such subjective assessments of IBS symptoms can be influenced by psychological factors.^38^ Thus, our findings may have been similarly affected. Variation in dosage regimens between the different RCTs also represents a potential confounding factor. The lowest total rifaximin dosage used was 800 mg/day, and the highest was 1650 mg/day. Although our data suggest a correlation between the OR of overall symptom relief and the total rifaximin dosage per day, no significant heterogeneity (*I*^2^ ≤ 50%) and no significant difference in effect size (*P* < 0.05) were observed for overall symptom relief at the end of the treatment and follow-up periods.

The use of different diagnostic criteria might also have influenced our findings. Three of the studies included in our meta-analysis used the Rome II diagnostic criteria,^24,25,27^ whereas the fourth study used the Rome I criteria.^26^ Furthermore, it is unclear whether our findings would have been similar based on the current Rome diagnostic criteria (Rome III). We know of only 1 study in India that has comprehensively compared the Rome I, II, and III criteria,^39^ and relatively few studies have directly compared the Rome I and II diagnostic criteria.^40,41^ In general, the Rome I and III criteria have been found to be more sensitive than those of Rome II in studies of both Asian and North American cohorts.^39,41,42^ By contrast, Dorn et al^43^ found that the Rome II and III diagnostic criteria were comparable for identifying IBS subtypes in a study in the USA. Therefore, no consensus exists for controlling for the effects of different versions of the Rome criteria in meta-analyses of RCTS of IBS treatments.

Nonetheless, our findings provide significant support for the use of rifaximin for treating IBS and warrant the undertaking of future RCTs examining the use of rifaximin with other antibiotics or probiotics, which have also previously demonstrated therapeutic effects on IBS,^44,45^ to determine whether the individual benefits of each might be additive when used in combination. Furthermore, our analysis of abdominal distention, which was based on the results of a single RCT with 191 IBS patients, indicates that the benefits of rifaximin treatment may be delayed in at least some patients, highlighting the need for the standardization of long-term follow-up endpoints in rifaximin trials to ensure that data for IBS clinical outcomes are not lost due to variation in patient response to rifaximin treatment.
